# Cumulative incidence of sickness absence and disease burden among the newly sick-listed, a cross-sectional population-based study

**DOI:** 10.1186/1471-2458-13-329

**Published:** 2013-04-10

**Authors:** Brynja Ármannsdóttir, Ann-Charlotte Mårdby, Inger Haukenes, Gunnel Hensing

**Affiliations:** 1 Department of Community Medicine and Public Health, The Sahlgrenska Academy, University of Gothenburg, Gothenburg, Sweden; 2 Department of Public Health and Primary Health Care, University of Bergen, Bergen, Norway

**Keywords:** Sickness absence, Incidence, Chronic disease, Gender, Socio-economic status

## Abstract

**Background:**

Sickness absence is a public health problem with economic consequences for individuals and society. Although sickness absence and chronic diseases are correlated, few studies exist concerning the role of chronic disease in all-cause sickness absence. The aim was to assess the cumulative incidence of sickness absence and examine the accompanying burden of chronic diseases among the sick-listed.

**Methods:**

A cross-sectional study was performed with data from 2008. Cumulative incidence of all-cause sickness absence (≥14 days) was calculated based on all newly sick-listed individuals (N = 12,543). The newly sick-listed sample and a randomized general population sample (n = 7,984) received a questionnaire (participation rates: 54% and 50%).To assess the burden of self-reported chronic diseases, standardized incidence ratios (SIR) were calculated.

**Results:**

Estimated one-year cumulative incidence was 11.3% (95% CI: 11.2–11.3), 14.0% (13.9–14.1) for women and 8.6% (8.5–8.6) for men. Gender differences were consistent across all age groups, with highest cumulative incidence among women aged 51–64 years, 18.2% (18.0–18.5). For women, the burden of chronic disease was significantly higher for nine out of twelve disease groups, corresponding numbers for men were nine out of eleven disease groups (standardized for age and socio-economic status). Neoplastic diseases had the highest SIR with 4.3 (3.4–5.2) for women and 4.2 (2.8–5.6) for men. For psychiatric and rheumatic diseases the respective SIR’s were 1.7 for women and 1.8 for men. The remaining disease groups had an elevated risk of 20-60% (SIR 1.2–1.6). The risk of reporting a co-morbidity was increased for women (SIR 1.4 (95% CI 1.4–1.5)) and men (SIR 1.5 (1.4–1.7)) among the sick-listed.

**Conclusions:**

Register data was used to estimate of the cumulative incidence of sickness absence in the general population. A higher burden of chronic disease among the newly sick-listed was found. Targeting long-term health problems may be an important public health strategy for reducing sickness absence.

## Background

Sickness absence in Sweden is higher than in other Northern European countries [1] possibly related to higher employment rates, particularly among women and older workers [2], thus including a larger proportion of employees with underlying health problems [3]. An OECD report from 2006 on sickness absence in Sweden stated an urgent need to address the “medicalisation” of the labour market [4]. This refers to the inclusion of individuals absent for other reasons than illness amongst the sick-listed, diverting focus from health towards other factors contributing to sickness absence. It has been proposed that high sickness absence in Sweden portrays concealed unemployment [5]. The level of sickness absence in Sweden is considered both a public health and an economic problem due to marginalization of individuals, benefit payments and production loss [6,7]. However, sickness absence is a multifactorial phenomenon, not only dependent on the current illness causing the sickness absence episode, but also various other factors such as the psychosocial and physical work environment, family support and the general health status of the individual [8-10]. Sickness absence can be seen as an integrated measure of physical, psychological, and social functioning [11]. With respect to gender, women in Sweden generally have a higher sickness absence rate compared to men [12]. This has not been seen in all countries, age groups or professions and seems more prominent in short-term absences [13,14]. Another important aspect is the social gradient observed in sickness absence, with higher rates for lower socio-economic groups [15,16]. Furthermore, sickness absence has been related to future adverse health outcomes, mortality and subsequent lower self-rated health for up to 14 years, irrespective of self-reported chronic conditions [17-19]. Other studies have found that sickness absence increases the future risk of disability pension [20,21] and that sick leave lasting longer than three weeks is associated with poor self-reported health, physical complaints, low mental well-being and poor work ability [22].

In light of the “medicalisation” of the labour market previously mentioned, surprisingly few studies have examined the health status of individuals with all-cause sickness absence. Chronic diseases and sickness absence appear to be correlated and chronic disease can predict long-term sickness absence and disability pension [23,24]. The presence of at least one long term disease predicts long term sickness absence [25,26] and the risk of sickness absence seems to be even higher for those with a co-morbid disease [24,27,28]. To decrease the rates of sickness absence it is important to acquire accurate information on the newly sick-listed employed population, which is at risk of proceeding into long term sickness absence. Correct information on the incidence of sickness absence as well as the health status of the sick-listed individuals is essential so that resource distribution and preventive measures can be focused on those at risk.

The aim of this study was to assess the cumulative incidence of sickness absence, and to estimate and compare the burden of chronic disease among the newly sick-listed and the general population. The hypothesis was that the burden of underlying chronic disease is higher among newly sick-listed individuals than the general population.

## Methods

The current study design was cross-sectional and part of the Health Assets Project (HAP) [29] initiated in 2008. The main purpose of HAP was to examine the influence of individual, organizational, and societal factors on health, sickness absence, and return to work. The study base was the population of Västra Götaland region in Sweden (approximately 1.6 million inhabitants, representing 17% of the Swedish population) and included both urban and rural areas.

The present study was divided into two parts. The first part was register-based including all employed individuals (n=12,543) who had one sick-leave spell ≥14 days, during the period 18^th^ of February to 15^th^ of April 2008. The second part of the study was questionnaire-based and examined the self-reported chronic diseases in a sample of the newly sick-listed and a random sample from the general population obtained with the help of Statistics Sweden. The newly sick-listed sample (n=6,140) consisted of all individuals who were *registered* as sick-listed at the Social Insurance during the inclusion period of February to 15^th^ of April 2008. The sample constitutes 49 % of those who became sick-listed during the period. The remaining 51% (n=6,403) became sick-listed during the time period but were, due to administrative reasons *registered* after the 15^th^ of April 2008. This group was not invited to the questionnaire study since we aimed at distributing the questionnaire as close as possible to the actual sick-leave period. The register study was based on official statistics and did not include any individual level data. Participation in the questionnaire study was based on informed consent. Both the register- and questionnaire-based parts of this study were approved by the Regional Ethical Review Board in Gothenburg, Sweden (Dnr: 039–08) and conducted in accordance with the latest version of the Helsinki protocol.

### Study population

To assess cumulative incidence the population at risk was used as denominator and consisted of all employed individuals aged 19–64 years (N = 668,887) living in the region (Table 1). All employed individuals who became sick-listed ≥ 14 days (N = 12,543) during the inclusion period constituted the numerator. Sickness absences lasting two weeks or less are employer-paid and therefore not registered by the Swedish Social Insurance Agency [30]. Denominator information was retrieved from Statistics Sweden and numerator information from the Swedish Social Insurance Agency.

**Table 1:** **Characteristics and socio**-**demographic data of the population at risk and the study samples**

	**Population at risk**		**Newly sick-listed population**		**General population sample**	
	**Men N = 336 085**	**Women N = 332 802**	**Men n = 2 386**	**Women n = 3 754**	**Men n = 4 086**	**Women n = 3 898**
Non-responders (percentage of sample)	^#^	^#^	1 272 (53)	1 558 (42)	2 293 (56)	1 664 (43)
Participants	^#^	^#^	n =1 113	n =2 196	n =1 793	n = 2 234
	N (%)	N (%)	n (%)	n (%)	n (%)	n (%)
Age						
19-30 years	83 474 (25)	76 588 (23)	129 (12)	251 (11)	366 (20)	464 (21)
31-50 years	163 719 (49)	162 391 (49)	445 (40)	1034(47)	802 (45)	1001 (45)
51-64 years	88 892 (26)	93 823 (28)	539 (48)	912 (42)	625 (35)	769 (34)
Socio-economic group						
Higher non-manual employees	°	°	114 (10)	245 (11)	328 (18)	308 (14)
Intermediate non-manual employees	°	°	169 (15)	597 (27)	369 (21)	574 (26)
Lower non-manual employees	°	°	73 (7)	315 (14)	159 (9)	365 (16)
Skilled worker	°	°	317 (28)	462 (21)	374 (21)	345 (15)
Unskilled worker	°	°	409 (37)	550 (25)	408 (23)	478 (21)
Other	°	°	0 (0)	0 (0)	64 (4)	55 (2)
Information on socio-economic status missing	°	°	31 (3)	28 (1)	91 (5)	109 (5)

^#^Inapplicable since questionnaire was not sent out to the entire population at risk. °Information on socio-economic status of the population at risk was not available.

In the second part of the study, 49 % of the newly sick-listed employed population (n = 6,140) and a random sample of the general population (n = 7,984) were invited to participate in a questionnaire study. Respondents to the questionnaire comprised the two samples, the newly sick-listed sample (n = 3,310) and the general population sample (n = 4,027). The characteristics and socio-demographic data of the population at risk, the newly sick-listed population as well as respondents to the questionnaire can be seen in Table 1.

The participation rate in the newly sick-listed sample was 54% (n = 3,310), yielding a response rate of 58% (n = 2,196) for women and 47% (n = 1,114) for men. The participation rate in the general population sample was 50% (n = 4,027), 55% (n = 2,234) for women and 45% (n = 1,793) for men. Non-response in both sample groups was more likely in the youngest age group (19–30 years), the lowest annual income group, ≤149,000 SEK, and amongst those born outside the Nordic countries.

### Data collection and outcome measures

A self-administered questionnaire was mailed by Statistics Sweden to the two samples. The questionnaire included items on socio-demographic factors, physical and mental health, sick-leave, working life, family conditions, life-events, leisure and lifestyle and is included as an additional file [see Additional file 1].

Burden of chronic diseases was assessed using the following question: “Do you have a persistent disease, problem or disability?” Response options were given as a check list of eleven different disease groups (cardiovascular, pulmonary, dermatologic, musculoskeletal, rheumatic, neurologic, psychiatric, endocrine, neoplastic, gastrointestinal and gynaecologic disease/problem/disability) as well as the alternatives “no” and “other”. Participants who replied “other” then had the opportunity to provide their own answer. These answers were categorized into the eleven different disease groups during data processing. The definition of chronic disease or chronic condition is not agreed upon in literature. According to the World Health Organization (WHO) chronic diseases have a long duration and usually slow progression [31]. The term chronic disease will be used when referring to the aforementioned disease groups, even though some of the conditions reported might not fall under the strictest definitions of chronic disease.

The participants were divided into three age groups 19–30, 31–50 and 51–64 years. Socioeconomic status was based on occupational position derived from Statistics Sweden register data and categorized according to their definitions [32]. Forty-nine individuals required manual reclassification into relevant socio-economic groups based on self-reported data, six individuals in the newly sick-listed sample were excluded due to missing information on occupational position and a total of 106 individuals in the newly sick-listed sample and 152 individuals in the general population sample were excluded due to missing or internally conflicting answers to the questions regarding chronic disease.

### Statistical analysis

Statistical analysis was performed using IBM SPSS Statistics version 19.0.0 and Microsoft Office Excel 2007. The two-month cumulative incidence of all-cause sickness absence was calculated as a fraction of the population at risk:

$$\begin{array}{c} \text{Cumulative}\;\text{Incidence}\\ \;=\frac{\text{Incident}\;\text{cases}\;\left(\text{February}\;18\;\mathrm{th}-\text{April}\;15\mathrm{th}\right)}{\text{Number}\;\mathrm{of}\;\text{employees}\;\mathrm{in}\;2008} \end{array}$$

The one-year cumulative incidence of all-cause sickness absence was then estimated by multiplying the two-month cumulative incidence by six. Confidence intervals (CI) were calculated separately for the two-month cumulative incidence and the estimated one-year cumulative incidence [33].

In the second part of the study results for chronic diseases in the two samples were presented as a proportion out of the sample for each disease group. Proportions were compared by calculating 95% CI for the difference in proportions [34]. Burden of chronic disease was assessed by calculating standardized incidence ratios (SIR), where observed number of cases reporting a chronic disease or condition in the sick-listed population was divided by the expected number of cases [35]. Standardization was performed by dividing both samples into corresponding age and socioeconomic groups. The expected number of cases was calculated for the sick-listed sample using proportions derived from the randomized general population sample results in each group [35]. If the SIR was 1.0 the incidence in the newly sick-listed sample equalled that of the general population. The 95% CI of SIR was calculated and a significant difference between the two samples was noted when the confidence interval did not include 1.0.

## Results

### Cumulative incidence

The cumulative incidence of all-cause sickness absence was 1.9% (95% CI 1.8-1.9) for the two-month period which gave an estimated one-year cumulative incidence of 11.3% (95% CI 11.2-11.3) (Table 2). The highest one-year cumulative incidence was 18.2% (95% CI 18.0–18.5) for women aged 51–64 years. The older age groups had a higher cumulative incidence of all-cause sickness absence in general. Women had a higher cumulative incidence than men across all age groups, most prominently in the age group 31–50 years, where women had 1.8 times higher cumulative incidence. The least gender difference was seen among the youngest age group, 19–30 year olds.

**Table 2:** **All**-**cause cumulative incidence of sickness absence among employed individuals aged 19**–**64 years**^#^

**Age groups**	**2-month cumulative incidence**			**Estimated 1-year cumulative incidence**		
	**All**	**Men**	**Women**	**All**	**Men**	**Women**
	**N = 668 887**	**N = 336 085**	**N = 332 802**	**N = 668 887**	**N = 336 085**	**N = 332 802**
	**% (95% C.I.)**	**% (95% C.I.)**	**% (95% C.I.)**	**% (95% C.I.)**	**% (95% C.I.)**	**% (95% C.I.)**
19-30	1.3 (1.3–1.4)	1.0 (1.0–1.1)	1.7 (1.6–1.8)	8.0 (7.8–8.1)	6.1 (5.9–6.3)	10.0 (9.8–10.3)
31-50	1.9 (1.9–2.0)	1.4 (1.3–1.4)	2.5 (2.4–2.5)	11.5 (11.4–11.6)	8.3 (8.1–8.4)	14.7 (14.5–14.9)
51-64	2.3 (2.2–2.4)	1.9 (1.8–2.0)	3.0 (2.9–3.2)	13.7 (13.6–13.9)	11.4 (11.1–11.6)	18.2 (18.0–18.5)
All ages	1.9 (1.8–1.9)	1.4 (1.4–1.5)	2.3 (2.3–2.4)	11.3 (11.2–11.3)	8.6 (8.5–8.6)	14.0 (13.9–14.1)

^#^1-year cumulative incidence was estimated by multiplying the 2 month cumulative incidence by six.

### Gender differences – self-reported chronic diseases

Among the sick-listed, endocrine disease, musculoskeletal problems and rheumatic disease were significantly more often reported by women than men (Table 3). Men reported having a cardiovascular disease more often than women. Women were more likely to report more than one chronic disease or problem compared to men (33.0% vs. 22.1%).

**Table 3:** **Proportion reporting each chronic disease group among the newly sick**-**listed and the general population**^#^

	**Men**			**Women**		
	**Newly sick listed n = 1 073 % (95% C.I)**	**General population n = 1 730 % (95% C.I)**	**Difference % (95% C.I.)**	**Newly sick listed n = 2 131 % (95% C.I)**	**General population n = 2 145 % (95% C.I)**	**Difference % (95% C.I.)**
Neoplastic disease	3.2 (2.1–4.2)	0.7 (0.3–1.1)	0.03 (0.01–0.04)*	4.1 (3.2-4.9)	0.9 (0.5–1.3)	0.03 (0.02–0.04)*
Endocrine	5.5 (4.1–6.9)	3.2 (2.4–4.1)	0.02 (0.01–0.04)*	8.2 (7.0–9.3)	5.2 (4.2–6.1)	0.03 (0.02–0.04)*
Psychiatric	8.4 (6.7–10.0)	4.1 (3.2–5.0)	0.04 (0.02–0.06)*	11.4 (10.0–12.8)	6.9 (5.8–8.0)	0.05 (0.03–0.06)*
Neurologic	4.1 (2.9–5.3)	2.4 (1.7–3.2)	0.02 (0.00–0.03)	4.3 (3.5–5.2)	3.3 (2.5–4.0)	0.01 (0.00–0.02)
Cardiovascular	16.8 (14.5–19.0)	9.8 (8.4–11.2)	0.07 (0.04–0.10)*	10.5 (9.2–11.8)	7.4 (6.3–8.5)	0.03 (0.01–0.05)*
Pulmonary	10.4 (8.6–12.3)	11.0 (9.6–12.5)	−0.01 (−0.03–0.02)	13.8 (12.3–15.3)	13.1 (11.7–14.5)	0.01 (−0.01–0.03)
Gastrointestinal	12.6 (10.6–14.6)	7.6 (6.3–8.8)	0.05 (0.03–0.07)*	15.4 (13.9–17.0)	12.1 (10.7–13.5)	0.03 (0.01–0.05)*
Skin and allergic	8.1 (6.5–9.7)	7.0 (5.8–8.2)	0.01 (−0.01–0.03)	10.5 (9.2–11.8)	10.7 (9.4–12.0)	0.00 (−0.02–0.02)
Musculoskeletal	21.5 (19.1–24.0)	14.4 (12.8–16.1)	0.07 (0.04–0.10)*	29.1 (27.2–31.0)	20.0 (18.4–21.7)	0.09 (0.07–0.12)*
Rheumatic	2.8 (1.8–3.8)	1.4 (0.8–2.0)	0.01 (0.00–0.03)	6.5 (5.5–7.6)	3.9 (3.1–4.7)	0.03 (0.01–0.04)*
Gynaecologic	–	–		5.0 (4.1–5.9)	3.9 (3.1–4.6)	0.01 (0.00–0.02)
Other	5.2 (3.9–6.5)	2.8 (2.0–3.5)	0.02 (0.01–0.04)*	3.3 (2.6–4.1)	2.6 (1.9–3.2)	0.01 (0.00–0.02)
No Disease	33.2 (30.4–36.0)	54.2 (51.8–56.5)	−0.21 (−0.25– -0.17)*	29.1 (27.2–31.1)	45.7 (43.6–47.8)	−0.17 (−0.19– -0.14)*
One chronic disease	44.7 (41.8–47.7)	32.6 (30.4–34.8)	0.12 (0.08–0.16)*	37.8 (35.8–39.9)	32.3 (30.3–34.3)	0.06 (0.03–0.08)*
More than one chronic disease	22.1 (19.6–24.6)	13.2 (11.6–14.8)	0.09 (0.06–0.12)*	33.0 (31.0–35.0)	22.0 (20.3–23.8)	0.11 (0.08–0.14)*

^#^Numbers are percentages with 95% confidence intervals in brackets. Significant difference between the two samples is marked with an asterisk (*).

In the youngest age group more women than men reported psychiatric disease and chronic gastrointestinal disease, reporting “no chronic disease” was more common among men than women. In the oldest age group (51–64 years) men reported having a chronic cardiovascular disease more often than women, while a larger proportion of women reported chronic musculoskeletal problems and rheumatic disease compared to men.

### Disease burden

**Figure 1:**
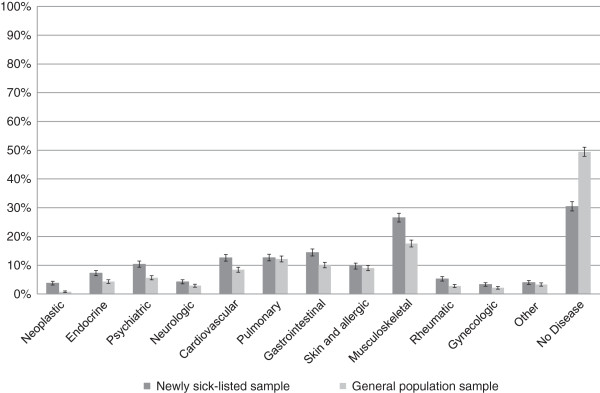
**Self**-**report of chronic diseases among the newly sick**-**listed and the general population.** Detailed legend: Proportion reporting each chronic disease group within the newly sick-listed sample and the general population sample are displayed. Error bars represent 95% Confidence intervals.

There was a significantly higher proportion reporting most chronic disease groups among the newly sick-listed sample compared with the general population sample (Figure 1). The chronic condition most commonly reported by both samples was musculoskeletal disorders (26.6% newly sick-listed, 17.1% general population). Neoplastic disease was reported nearly four times more often among the newly sick-listed (3.8% newly sick-listed, 0.8% general population). Rheumatic disease and psychiatric disease were reported almost twice as often by the newly sick listed sample (rheumatic disease: 5.3% newly sick listed, 2.8% general population; psychiatric disease: 10.4% newly sick listed, 5.5% general population). The proportion reporting no chronic disease among the newly sick-listed population was 30.5%, while the corresponding figure for the general population was 48.1%. Co-morbidities were more commonly reported by the newly sick-listed sample compared with the general population sample (29.4% newly sick listed, 18.1% general population).

When standardized for age, men and women in the newly sick-listed sample had a significantly higher burden of disease compared with the general population (Table 4). Women had a significantly higher burden of disease for eight out of the twelve chronic disease groups while the corresponding number for men was nine out of the eleven groups (excluding gynaecological disease).

**Table 4:** **Burden of chronic disease among the newly sick**-**listed compared to the general population sample**^#^

	**Standardized by age groups**						**Standardized by age and socio-economic groups**						**Standardized by age, socio-economic groups and gender**		
	**Men**			**Women**			**Men**			**Women**			**Total sample**		
	**Expected number of cases**	**Observed number of cases**	**Standardized incidence ratio (95% C.I)**	**Expected number of cases**	**Observed number of cases**	**Standardized incidence ratio (95% C.I)**	**Expected number of cases**	**Observed number of cases**	**Standardized incidence ratio (95% C.I)**	**Expected number of cases**	**Observed number of cases**	**Standardized incidence ratio (95% C.I)**	**Expected number of cases**	**Observed number of cases**	**Standardized incidence ratio (95% C.I)**
Neoplastic	9	34	4.0 (2.7–5.3)*	20	87	4.3 (3.4–5.2)*	8	33	4.2 (2.8–5.6)*	20	85	4.3 (3.4–5.2)*	1491	977	0.7 (0.6–0.7)*
Endocrine	41	59	1.4 (1.1–1.8)*	120	174	1.5 (1.2–1.7)*	38	58	1.5 (1.2–1.9)*	118	174	1.5 (1.3–1.7)*	314	403	1.3 (1.2–1.4)*
Psychiatric	44	90	2.0 (1.6–2.4)*	148	243	1.6 (1.5–1.8)*	41	87	2.1 (1.7–2.6)*	140	241	1.7 (1.5–1.9)*	386	406	1.1 (1.0–1.1)*
Neurologic	27	44	1.6 (1.2–2.1)*	73	92	1.3 (1.0–1.5)	26	42	1.6 (1.1–2.1)*	70	92	1.3 (1.1–1.6)*	297	311	1.0 (0.9–1.2)
Cardiovascular	130	180	1.4 (1.2–1.6)*	184	223	1.2 (1.1–1.4)*	124	173	1.4 (1.2–1.6)*	180	221	1.2 (1.1–1.4)*	642	851	1.3 (1.2–1.4)*
Pulmonary	109	112	1.0(0.9–1.2)	278	294	1.1 (0.9–1.2)	108	110	1.0 (0.8–1.2)	274	293	1.1 (1.0–1.2)	108	169	1.6 (1.3–1.8)*
Gastrointestinal	87	135	1.5 (1.3–1.8)*	255	328	1.3 (1.2–1.4)*	81	131	1.6 (1.4–1.9)*	249	325	1.3 (1.2–1.4)*	100	136	1.4 (1.1–1.6)*
Skin and allergic	76	87	1.1 (0.9–1.4)	221	224	1.0 (0.9–1.1)	72	84	1.2 (0.9–1.4)	223	222	1.0 (0.9–1.1)	192	333	1.7 (1.6–1.9)*
Musculoskeletal	174	231	1.3 (1.2–1.5)*	467	620	1.3 (1.2–1.4)*	166	228	1.4 (1.2–1.5)*	456	615	1.3 (1.3–1.4)*	161	233	1.4 (1.3–1.6)*
Rheumatic	17	30	1.7 (1.1–2.4)*	91	139	1.5 (1.3–1.8)*	17	30	1.8 (1.2–2.4)*	83	139	1.7 (1.4–2.0)*	29	121	4.2 (3.5–4.9)*
Gynaecologic	–	–	–	80	106	1.3 (1.1–1.6)*	–	–	–	79	105	1.3 (1.1–1.6)*	343	463	1.4 (1.2–1.5)*
Other	32	56	1.7 (1.3–2.2)*	57	71	1.2 (1.0–1.5)	32	54	1.7 (1.2–2.1)*	55	70	1.3 (1.0–1.6)	80	106	1.3 (1.1–1.6)*
No Disease	552	356	0.6 (0.6–0.7)	939	621	0.7 (0.6–0.7)	542	347	0.6 (0.6–0.7)	940	613	0.7 (0.6–0.7)	1563	977	0.6 (0.6–0.7)*
One chronic disease	364	480	1.2 (1.2–1.4)*	696	806	1.2 (1.1–1.2)*	357	469	1.3 (1.2–1.4)*	683	795	1.2 (1.1–1.2)*	1033	1286	1.2 (1.2–1.3)*
More than one chronic disease	157	237	1.5 (1.3–1.7)*	496	704	1.4 (1.3–1.5)*	150	230	1.5 (1.4–1.7)*	484	700	1.4 (1.4–1.5)*	607	941	1.5 (1.5–1.6)*

^#^Standardized incidence ratios (SIR) for chronic disease in the newly sick-listed sample are shown. If the SIR is 1.0 the incidence among the newly sick-listed equals that of the general population sample. A significant difference (*) between the two samples is observed when the 95% confidence interval does not include 1.0.

When standardized for age and socio-economic status, the burden of disease among the newly sick-listed compared with the general population was significantly higher for nine out of twelve chronic disease groups for women and nine out of eleven for men. Among the more common medical conditions the burden of disease (SIR) for musculoskeletal disorders was 1.3 for women and 2.4 for men; gastrointestinal 1.3 for women and 1.6 for men; psychiatric 1.7 for women and 2.1 for men; and cardiovascular 1.2 for women and 1.4 for men.

Among the less common diseases the highest SIR was for neoplastic disease (SIR 4.3 for women and 4.2 for men) followed by rheumatic disease (SIR 1.7 for women and 1.8 for men), endocrine disease (SIR 1.5 for women and 1.5 for men) and neurologic disease for men (SIR 1.6). The newly sick-listed had 40-50% higher risk of co-morbidity compared with the general population (SIR 1.4 for women and 1.5 for men) and were less likely to report no chronic disease (SIR 0.7 for women and SIR 0.6 for men).

## Discussion

This study offers an assessment of the cumulative incidence of all-cause sickness absence as well as the burden of chronic disease amongst the same individuals. The results of men and women differed in all age groups and chronic diseases were more prevalent amongst the sick listed than in the general population.

Information on incidence of sickness absence is important to grasp the size of the problem in terms of number of individuals involved, especially when following the development of sickness absence or planning preventive measures [36]. Previous research has often focused on self-reported sickness absence, sickness absence duration or on certain groups of employees or diseases, thereby portraying different aspects of the problem, not always easy to compare to other results.

The one-year cumulative incidence in this study was 11.3%, meaning that 75,600 individuals in Västra Götaland region would be sick-listed for a period lasting ≥14 days at least once annually. The cumulative incidence of sickness absence was studied in Sweden and Norway in the 1980’s and the 1990’s [37,38]. However, changes in both society and social insurance have taken place since then making comparisons difficult [39]. A study by Lidwall and Marklund (2010) showed great variation in the number of ongoing sickness absence cases over the years 1992–2008, underscoring the inherent difficulty in comparing different periods [40]. Eriksson et al. (2008) found that 40% of workers reported any sickness absence during the previous year, while only 4% had been absent over 29 days, a figure comparable to ours [41]. Findings from Denmark show that the cumulative incidence of sickness absence (also of a minimum of two weeks) among employed people increased from 6.6% to 7.5% between 2000 and 2007, which is slightly lower than our findings [42].

Our results show that the cumulative incidence of sickness absence increased in the older age groups approaching retirement for both genders. This is not unexpected since medium and long term sickness absence is more common in older age group. Sweden has high employment rates overall and in older age groups [2], which means that more employees with health problems might be present in the workforce compared to other countries [3]. This can be one reason for the higher sickness absence levels in Sweden. The ageing of the population and the trend towards a higher retirement age may also lead to increased numbers of individuals with chronic disease in the workforce [43].

With respect to the gender difference by age, the cumulative incidence among women increased stepwise between age groups, whereas amongst men the most prominent difference was between the middle and the oldest age groups. A Norwegian study found that women <50 years of age and sick-listed >8 weeks due to musculoskeletal disorders had a higher risk of chronicity than men in the same age group [44]. The authors suggest that women may develop chronic musculoskeletal disorders at an earlier age than men. With respect to the current study, the substantial increase in cumulative incidence of sickness absence among women aged 31–50 years compared with men in the similar age group may be influenced by the phenomenon of earlier chronicity. Moreover, women in this particular age-group are often engaged in both work and family. According to the double burden hypothesis, women combining careers with responsibility for children and domestic work may face a higher risk of sickness absence [45]. In a Swedish longitudinal study women taking on a parental role during follow up had increased odds for sickness absence compared to those not adding such a role [46]. Other studies have also linked having young children to increases in sickness absence for women, however this has also been seen for men [13].

The labour market in Sweden is highly gender segregated. Women and men tend to work in different sectors (horizontal segregation) and men tend to have higher positions in the workplace and the occupational hierarchy (vertical segregation). Both horizontal and vertical segregation seem to influence sickness absence rates [13,47]. A Finnish study found that the overall gender differences in sickness absence can be explained by the fact that it is more common that women have shorter absences [48]. This does not seem to apply to our results as the gender difference was apparent despite exclusion of shorter absence periods.

In the current study, gender differences in relation to the different self-reported chronic disease groups were in line with what has been observed previously in Sweden (age group 35–64 years), where chronic musculoskeletal, gastrointestinal and psychiatric symptoms as well as rheumatoid arthritis, were more prevalent among women [49]. Significantly more men reported having cardiovascular disease than women, correlating with the fact that women have been found to develop cardiovascular disease 7–10 years later in life than men [50]. The importance of chronic disease as a contributory factor to gender differences has not been much explored. A Swedish study proposes a triple burden for women and men with chronic disease added to the demands experienced from paid and unpaid work [51]. Women more often than men experience a double burden in daily life. The presence of chronic disease as a third burden may render women more vulnerable to the work-related burden and place them at increased risk for sickness absence.

In general, the newly sick-listed reported more chronic disease and were more likely to have co-morbid diseases than the random general population sample. The most commonly reported chronic diseases in the sick-listed group were musculoskeletal, gastrointestinal, psychiatric and cardiovascular diseases. Studies have suggested that complex symptoms such as musculoskeletal pain, digestive problems and mental problems are the leading causes of sickness absence [52]. These conditions display different symptoms, may appear separately or in combination and may share a common mechanism of origin. In the current study, the high burden of musculoskeletal, gastrointestinal, psychiatric and co-morbid conditions among the sick-listed may be interpreted with this in mind. The higher disease burden when compared with the general population remained consistent throughout standardisation for age and socio-economic status. There are few studies examining the burden of sickness absence among individuals with chronic disease. A study on individuals with angina pectoris found an almost threefold higher rate of sickness absence compared with a sample not suffering from chronic disease [24]. Similar results have also been reported for fibromyalgia and asthma [27,28].

There seems to be an even higher risk when more than one chronic disease is present [24,27,28]. In the current study we found a substantially increased risk of having co-morbid diseases among the newly sick-listed compared with the general population. The reporting of neoplasia was nearly four times higher which might reflect that most individuals diagnosed with a malignancy become sick-listed. This might be due to severe physical symptoms and the difficult treatments together with the psychological and social impacts associated with this disease [53]. Sick-listed men and women had an increased risk of reporting psychiatric disease, rheumatic disease and endocrine disease and sick-listed men had a substantially higher risk of reporting neurologic disease. These findings show that the burden of chronic diseases seemed to be significantly higher among newly sick-listed individuals compared with the general population, and that this difference was not explained by a different composition of the newly sick-listed population when it comes to sex, age or socio-economic status. It is also noteworthy that this result held true when standardized for socio-economic status in addition to age compared to standardizing for age alone indicating that those with a chronic disease are more likely to become sickness absent, regardless of socio-economic class.

### Strengths and weaknesses

A major strength of the current study was the inclusion of all newly sick-listed individuals with employment providing a population based estimate of the cumulative incidence of sickness absence. Demographic information and socio-economic status were derived from highly reliable Statistics Sweden register data. A possible weakness in our methodology is an underestimation of the cumulative incidence if part of the working population was not at risk, possibly due to sickness absence.

The estimation of the one-year cumulative incidence might be subject to error due to seasonal variation. Sickness absence rates are lower during the second and third quarters and higher in the first and fourth quarters [54]. The sickness absence cases included in this study were spread over the second part of the first quarter and the first half of the second quarter. It is therefore likely that the effects of seasonal variation in the current study were minimized. A study from 1974 addressed seasonal variation in all-cause sickness absence specifically and found that most seasonal variation was due to upper respiratory disease and bronchitis in addition to digestive disorders [55]. This strengthens the assumption that the effects of seasonal variation in this study ought to have been minimal as sickness absence due to respiratory infections or gastroenteritis rarely exceeds two weeks [56] and would therefore not have affected our results.

Data in the second part of the study is based on self-reported data and there are several potential causes for bias. There was a drop-out in the first phase of participant inclusion. Not all individuals sick-listed between the 18^th^ of February and the 15^th^ of April 2008 were formally registered during the period and included in our study. The delay was primarily for administrative reasons, such as determination of income in order to calculate the appropriate sickness benefit. In our study we nevertheless include 49% of the sick-listed during the period, which is a large sample size. The group with later registration had a higher proportion of individuals with low income, of men (male/female ratio 1.32), of highly educated and first time sick-listed as well as a slight overrepresentation of immigrants (immigrant/non-immigrant ratio 1.08) according to our correspondence with the Department for Statistics and Analysis at the Swedish Social Insurance Agency. Unfortunately more detailed statistical information is unavailable at this time from the agency. Thus no information is available regarding any difference in the diagnostic groups dependent on the time of registration. Therefore there is a possibility for a systematic bias based on differences in registration date which we cannot completely exclude. This risk is somewhat offset by the large sample size but the smaller diagnostic groups (for example neoplastic disease) would be more sensitive to any such error.

Another possible source of bias is the relatively low response rate which may have led to a selection bias. The non-response rate was highest in the youngest age group, those with the lowest income and those born outside of the Nordic countries. The results are therefore less applicable to those groups. The results should also be interpreted in the light that the selection bias could also be affected by other variables not captured by the non-response analyses from Statistics Sweden. Information on chronic diseases and conditions was based on self-reported data so recall and reporting bias may therefore have occurred. Those who were sick-listed or had recently been sick-listed may have been more prone to remembering or reporting chronic diseases. This could have led to an overestimation of the standardized incidence ratios. A health response bias might also be present, with the healthiest individuals responding. Sensitivity and specificity of self-reported data on chronic disease has been found to vary from one disease group to another, with data for cardiovascular disease, diabetes and cancer being most reliable [57,58] while data for musculoskeletal [58] and psychiatric [59] disorders is less reliable [58]. Musculoskeletal disorders had the highest frequency in both samples and even if all of these might not be confirmable with clinical examination, these results give insight into the subjective experience of musculoskeletal problems among the sick-listed and the general population. The frequencies of psychiatric disorders in both samples are lower than the 15-25% 12 month prevalence of mental disorders reported by the WHO [60]. This may be due to responders being less likely to disclose mental health problems due to stigma and the results should be interpreted with the possibility of underestimation of psychiatric problems in mind.

Finally, the newly sick-listed sample was composed solely of employed individuals while the general population sample was a random sample of the total population. While we have corrected for the differences in age and socioeconomic position between the groups with standardisation for these factors, we cannot however rule out the possibility of other variations between the groups, some of which might affect our results. This is a possible major limitation of our study, which is however difficult to avoid when seeking a comparison group for the sick-listed population. An example of this type of effect is that we may have underestimated the differences between the newly sick-listed employed population and the general population as the employed population was a healthier group than the general population, which included the unemployed, individuals on disability pension as well as individuals on recurrent or long-term sick-leave.

## Conclusion

The current study gives an estimate of cumulative incidence of sickness absence among the working population in western Sweden. Our results indicate that the newly sick-listed working population had a higher rate of frequently reported chronic diseases, such as musculoskeletal, gastrointestinal, mental and cardiovascular disorders than the general population. The study results also show important age-related gender differences in sickness absence and burden of disease, and points to women’s multiple roles in society as one potential explanation. The study highlights the importance of taking health status into account when designing preventive interventions. Targeting these long-term health problems may be an important factor in any attempt to decrease sickness absence.

## Competing interests

The authors declare that they have no competing interests.

## Authors’ contributions

The study was conceived of and designed collaboratively by all the authors. BÁ, ACM, IH and GH interpreted the data. BÁ performed the statistical analysis and drafted the manuscript which was revised by BÁ, ACM, IH and GH. All authors read and approved of the final manuscript.

## Pre-publication history

The pre-publication history for this paper can be accessed here:

http://www.biomedcentral.com/1471-2458/13/329/prepub

## Supplementary Material

Additional file 1 **An English translation of the self-administered questionnaire from the HAP study.** This questionnaire was sent to both the newly sick-listed population and the randomized general population sample. For this study we primarily utilized the responses to question number 7. Click here for file
