# Supplementary material for: Cumulative incidence of sickness absence and disease burden among the newly sick-listed, a cross-sectional population-based study
Source: BMC Public Health. 2013 Apr 10;13:329. doi: 10.1186/1471-2458-13-329 (PMC3626677; doi:10.1186/1471-2458-13-329)
Supplement: Additional file 1 — An English translation of the self-administered questionnaire from the HAP study. This questionnaire was sent to both the newly sick-listed population and the randomized general population sample. For this study we primarily utilized the responses to question number 7. [file 1471-2458-13-329-S1.pdf]

A. Background, Education and Finances

1. **Are you a man or a woman?**

1 ☐ Man
2 ☐ Woman

2. **What is your marital status?**
Note! Mark only one alternative
1 ☐ Single
2 ☐ Married/partner with a man
3 ☐ Married/partner with a woman
4 ☐ In a relationship with a man but living separately
5 ☐ In a relationship with a woman but living separately
6 ☐ Widow/widower

3. **What is your year of birth?**

Year of birth: 1 9 ☐ ☐

4. **What is the highest level of education that you have completed?**
Note! Mark only one alternative
1 ☐ Compulsory schooling, elementary school, junior secondary school or similar
2 ☐ 2 years upper secondary school education or vocational training school
3 ☐ 3 or 4 years upper secondary school education
4 ☐ University or higher education studies, less than 3 years
5 ☐ University or higher education studies, 3 years or longer
6 ☐ Did not finish compulsory education or equivalent obligatory schooling

5. **Approximately, how much is your household's total monthly income before tax?**
Count the total income for everyone in the household! Income includes salary, unemployment benefit, benefit from Social Insurance Office (Försäkringskassan), operating income, pensions etc. as well as various allowances, such as child allowance, study grant and maintenance.
1 ☐ 0 – 14,999 SEK
2 ☐ 15,000 – 29,999 SEK
3 ☐ 30,000 – 44,999 SEK
4 ☐ 45,000 – 59,999 SEK
5 ☐ 60,000 SEK or more

B. Health

6. **In general, I would say that my health is**

1 ☐ Excellent
2 ☐ Very good
3 ☐ Good
4 ☐ Not so good
5 ☐ Poor

7. **Do you have any lasting illness, health problem or handicap?**
.Check all that apply.

1 ☐ No
1 ☐ Cardiovascular disease, abnormal blood pressure
1 ☐ Asthma/bronchial problems/allergy
1 ☐ Dermatitis/eczema/allergy
1 ☐ Symptom/pain in muscles, joints, connective tissue
1 ☐ Rheumatic disease
1 ☐ Neurological illness
1 ☐ Mental problems
1 ☐ Endocrinological disease (e.g. diabetes, goitre)
1 ☐ Tumour
1 ☐ Stomach problem
1 ☐ Gynaecological problems
1 ☐ Another illness

State which, write in the box: ☐

8. How tall are you?

☐☐☐ cm

9. How much do you weigh?

☐☐☐ kg

10. How often have you had the following symptoms during the past 12 months?

Mark one box in each row with an 'x'.

		Nearly every day	Now and again during the week	Now and again during the month	Almost never or never
		1	2	3	4
a.	Stomach problem	☐	☐	☐	☐
b.	Heart palpitations	☐	☐	☐	☐
c.	Difficulty breathing	☐	☐	☐	☐
d.	Fatigue	☐	☐	☐	☐
e.	Dizziness	☐	☐	☐	☐
f.	Headache	☐	☐	☐	☐
g.	Chest pain	☐	☐	☐	☐
h.	Back pain, backache, sciatica	☐	☐	☐	☐
i.	Neck pain and/or shoulder pain	☐	☐	☐	☐
j.	Difficulty getting to sleep	☐	☐	☐	☐
k.	Waking up frequently and difficulty getting back to sleep	☐	☐	☐	☐
l.	Difficulty concentrating	☐	☐	☐	☐

11. How have you felt during the past week?

Place an 'x' in the box that agrees best with each statement.

		All the time<br>1	Often<br>2	Sometimes<br>3	Never<br>4
a.	I have felt sad and down	☐	☐	☐	☐
b.	I have felt calm and relaxed	☐	☐	☐	☐
c.	I have felt energetic, active and go-ahead	☐	☐	☐	☐
d.	When I woke up, I felt alert, rested and full of enterprise	☐	☐	☐	☐
e.	I have felt happy or pleased and satisfied with my personal life	☐	☐	☐	☐
f.	I feel satisfied with my life situation	☐	☐	☐	☐
g.	I am living the kind of life I want to live	☐	☐	☐	☐
h.	I have been keen to deal with the day's work or to make new decisions	☐	☐	☐	☐
i.	I have felt that I can cope with serious problems or changes in my life	☐	☐	☐	☐
j.	I have felt that life is full of interesting things	☐	☐	☐	☐

12. How much do you agree with the following statements?

Mark one box in each row with an 'x'.

		Agree totally<br>1	Agree rather well<br>2	Do not agree particularly well<br>3	Do not agree at all<br>4
a.	I always succeed in solving difficult problems, if I just try hard enough	☐	☐	☐	☐
b.	Even if someone works against me, I still find a way to achieve my goals	☐	☐	☐	☐
c.	I do not have any difficulty sticking to my goals and accomplishing my goals	☐	☐	☐	☐
d.	In unexpected situations, I always know how I will act	☐	☐	☐	☐
e.	Even in surprising situations, I believe I can cope well.	☐	☐	☐	☐
f.	Thanks to my own ability, I feel calm, even when I have to face difficulties	☐	☐	☐	☐
g.	Whatever happens, I always cope	☐	☐	☐	☐
h.	I can always find a solution, no matter what problem I have to face	☐	☐	☐	☐
i.	If I have to face new challenges, I know how I will handle them	☐	☐	☐	☐
j.	When problems arise, I can normally handle them by my own efforts	☐	☐	☐	☐

13. Have you at any time felt so mentally ill that you felt the need to seek treatment?

1 ☐ Yes

2 ☐ Yes, but I didn't seek treatment → *Go to question 16*
3 ☐ No → *Go to question 17*

14. When you felt so mentally ill that you needed to seek treatment, where did you seek treatment?

1 ☐ Health centre
2 ☐ Open psychiatric care
3 ☐ With a private doctor
4 ☐ With a private psychologist or psychotherapist
5 ☐ Casualty department

6 ☐ Other

State where, write in the box: ☐

15. Do you think you received the treatment you needed?

1 ☐ Yes → ***Go to question 17***
2 ☐ No → ***Go to question 17***

16. What was the reason for you not seeking treatment?

Select all that apply.

1 ☐ I didn't believe that treatment could help me
1 ☐ I was ashamed to show that I felt so bad
1 ☐ I was afraid that someone I knew would see me when I sought treatment.
1 ☐ The travel time to the care provider was too long
1 ☐ The care provider was not open
1 ☐ It was too expensive to seek treatment
1 ☐ I didn't know where to turn
1 ☐ The wasn't any means of transportation, so I couldn't get to the care provider
1 ☐ I thought the mental illness was sure to disappear by itself
1 ☐ I was afraid I would be admitted against my will

1 ☐ Other reason

State the reason, write it in the box: ☐

C. Sick Leave and Sick Pay

17. Have you been on sick leave on one or more occasions during the past 12 months?
If you are on sick leave at the moment, do not count the current period of sick leave.
Count the total number of days, including weekends and holidays.

1 ☐ No
2 ☐ Yes →
1 ☐ 1 - 7 days
2 ☐ 8 - 30 days
3 ☐ 2 – 3 months
4 ☐ 4 – 12 months

18. **During the past 8 weeks, have you started a period of sick leave that has continued for more than 14 days?**

1 ☐ No
2 ☐ Yes

19. **Are you on sick leave at the moment?**

1 ☐ No ⟶ *If No, **go to question 25***
2 ☐ Yes

20. **What percentage of time are you on sick leave?**
If you are working, what is the percentage of your normal work time during which you are on sick leave?

1 ☐ 0 - 25 %
2 ☐ 26 - 50 %
3 ☐ 51 - 75 %
4 ☐ 76 - 100 %

21. **How long have you been on sick leave?**
Count from the first day of absence due to illness for the current period of sick leave. Count all the days of the week, including weekends and holidays.

☐☐☐ days

22. **What is the reason for you being on sick leave, according to your medical certificate**
Select as many as apply.

1 ☐ Infectious illness (e.g. cold, influenza)
1 ☐ Backache
1 ☐ Pain in the neck or shoulders
1 ☐ Other muscle or joint symptom/pain
1 ☐ Depression
1 ☐ Stress
1 ☐ Anxiety, worry
1 ☐ Schizophrenia or another psychotic illness
1 ☐ Another mental illness
1 ☐ Cardiovascular disease (e.g. heart attack, stroke, high blood pressure)
1 ☐ Problems during pregnancy
1 ☐ Injury/accident
1 ☐ Another illness
State which, write in the box: ☐

23. When do you think you will be at work full time/not on sick leave again?

1 ☐ Within 1 week
2 ☐ Within 2-3 weeks
3 ☐ Within 1-2 months
4 ☐ Within 3-6 months
5 ☐ Within 7-12 months
6 ☐ After 12 months
7 ☐ Never
8 ☐ Do not know

24. To what degree do you feel shame in front of others because you are on sick leave?

1 ☐ To a very high degree
2 ☐ To a high degree
3 ☐ Partly
4 ☐ To a slight degree
5 ☐ To a very slight degree

25. Are you receiving sick pay or activity benefit at the moment?

1 ☐ No → If No, **go to question 28**
2 ☐ Yes

26. What percentage of sick pay or activity benefit do you receive?

1 ☐ 0 - 25 %
2 ☐ 26 - 50 %
3 ☐ 51 - 75 %
4 ☐ 76 - 100 %

27. How long have you received sick pay or activity benefit?
Count from the first day of the current period of sick pay or activity benefit. Count all the days of the week, including weekends.

☐☐☐ days

D. Working Life

28. **What is your main occupation just now?**
If you are on sick leave, enter what your main occupation was before you went on sick leave. Note! Mark only one alternative

1 ☐ Work as an employee
2 ☐ Self employed
3 ☐ Studying
4 ☐ Sickness or activity benefit, early retirement pension
5 ☐ Parental leave
6 ☐ In search of work or in employment measures
7 ☐ Work in the home, look after the household

8 ☐ Other, write in the box:

29. **a. What is/was you main occupation?**

If you are not working at the moment, state the main occupation you have had.
Try to provide as detailed a job title as possible.
For example: Instead of assistant, write purchasing assistant. ***Please print!***
Example: Instead of driver, write:

B	U	S	S	C	H	A	U	F	F	Ö	R							

Your occupation:

b. What are/were you main work tasks?

Describe your main work tasks. For example, if you are a project leader or similar, then write what you do, for instance, "responsible for improving the work environment in geriatric care". If you are a factory worker, write what you do/manufacture.

The following questions concern your work. If you are on <u>part-time sick leave</u>, reply based on what it is like at the moment. If you are on <u>full-time sick leave</u> answer the questions based on what it was like at your work before you went on sick leave. If you have two or more jobs, answer based on your main job.

If you are <u>unemployed, receiving sick pay or activity benefit, or are not working for another reason</u>, go to section E.

30. Do you have a managerial/supervisory post?

1 ☐ No
2 ☐ Yes ——→ If Yes, how many people do you supervise? ☐☐☐

31. What is your main form of employment?
Note! Mark only one alternative

1 ☐ Permanent job (until further notice)
2 ☐ Employed for a specific project
3 ☐ Stand-in
4 ☐ Employed by the hour
5 ☐ Self employed
6 ☐ Another form of employment
State which, *write in the box:*

32. Do you work full-time or part-time

1 ☐ Full-time
2 ☐ Part-time, at least 15 hours a week
3 ☐ Part-time, less than 15 hours a week

33. b. Who is you main employer?
Note! Mark only one alternative

1 ☐ Privately-owned company
2 ☐ The government (authority/agency/state owned company)
3 ☐ Region/County Council/County Council-owned company
4 ☐ Municipality/Municipally-owned company
5 ☐ Self employed
6 ☐ Other
State which, *write in the box:*

34. What is the distribution according to sex where you primarily perform your daily work?

1 ☐ They are mainly women
2 ☐ There are roughly as many women as men (i.e. 20 - 60 percent of each sex)
3 ☐ They are mainly men
4 ☐ I work alone

35. Has your workplace been reorganised during the past 12 months?

1 ☐ Yes
2 ☐ No

36. Does your work require a large amount of concentration?

1 ☐ Yes, frequently
2 ☐ Yes, sometimes
3 ☐ No, rarely
4 ☐ No, never/almost never

37. Does your work require you to remember many things?

1 ☐ Yes, frequently
2 ☐ Yes, sometimes
3 ☐ No, rarely
4 ☐ No, never/almost never

38. Does your work require heavy lifting?

1 ☐ Yes, frequently
2 ☐ Yes, sometimes
3 ☐ No, rarely
4 ☐ No, never/almost never

39. Do you work in a crooked, twisted or otherwise unsuitable working posture?

1 ☐ Yes, frequently
2 ☐ Yes, sometimes
3 ☐ No, rarely
4 ☐ No, never/almost never

40. How do you judge your current capacity to work with respect to:
Mark with an 'x' in each row.

		Very good<br>1	Reasonably good<br>2	Not so good<br>3	Rather poor<br>4	Very poor<br>5
a.	…the knowledge demanded by the job?	☐	☐	☐	☐	☐
b.	…the mental and psychological demands of the job?	☐	☐	☐	☐	☐
c.	…the emotional demands of the job?	☐	☐	☐	☐	☐
d.	…the demands for cooperation required by the job?	☐	☐	☐	☐	☐
e.	…the physical demands of the job?	☐	☐	☐	☐	☐

41. When the work becomes *physically troublesome*, do you have the possibility of slowing down or of working differently, so the problem is reduced?

1 ☐ Yes, frequently
2 ☐ Yes, sometimes
3 ☐ No, rarely
4 ☐ No, never/almost never

42. **When the work becomes *mentally stressful*, do you have the possibility to influence what you work with, so the stress is reduced?**

1 ☐ Yes, frequently
2 ☐ Yes, sometimes
3 ☐ No, rarely
4 ☐ No, never/almost never

43. **Do you feel rested and restored when you start work?**

1 ☐ Yes, frequently
2 ☐ Yes, sometimes
3 ☐ No, rarely
4 ☐ No, never/almost never

44. **What is your work like?**
Mark with a cross on each row.

		Yes, frequently 1	Yes, sometimes 2	No, rarely 3	No, never 4
a.	Does your work require you to work very fast?	☐	☐	☐	☐
b.	Does your work require you to work very hard?	☐	☐	☐	☐
c.	Does your work require too much effort?	☐	☐	☐	☐
d.	Do you have sufficient time to keep up with your work tasks?	☐	☐	☐	☐
e.	Do conflicting requirements arise in your work?	☐	☐	☐	☐
f.	Are you allowed to learn new things in your work?	☐	☐	☐	☐
g.	Does your work require skill?	☐	☐	☐	☐
h.	Does your work require ingenuity?	☐	☐	☐	☐
i.	Does your work mean that one does the same thing time and time again?	☐	☐	☐	☐
j.	Are you free to decide how your work is to be performed?	☐	☐	☐	☐
k.	Are you free to decide what is to be done in your work?	☐	☐	☐	☐

45. **How well do you agree with the following statements?**
Mark with an ‘x in each row. If you do not have any workmates, go to question 46.

		Agree totally	Agree rather well	Do not agree particularly well	Do not agree at all
		1	2	3	4
a.	There is a calm and agreeable atmosphere at my place of work	☐	☐	☐	☐
b.	There is good harmony	☐	☐	☐	☐
c.	My workmates stand by me	☐	☐	☐	☐
d.	They understand that I can have a bad day	☐	☐	☐	☐
e.	I get on well with my superiors	☐	☐	☐	☐
f.	I get on well with my workmates	☐	☐	☐	☐

46. **Do you feel committed to your work?**

1 ☐ To a very high degree
2 ☐ To a high degree
3 ☐ Somewhat
4 ☐ To a slight degree
5 ☐ To a very slight degree

47. **Do you make high demands of yourself in your work?**

1 ☐ Yes, frequently
2 ☐ Yes, sometimes
3 ☐ No, rarely
4 ☐ No, never/almost never

48. **Do you find it difficult to say no to tasks, even though you already have a lot to do?**

1 ☐ Yes, frequently
2 ☐ Yes, sometimes
3 ☐ No, rarely
4 ☐ No, never/almost never

49. **Do you take more responsibility for the work than you should do?**

1 ☐ Yes, frequently
2 ☐ Yes, sometimes
3 ☐ No, rarely
4 ☐ No, never/almost never

50. Does your immediate manager pay attention to your opinions?

1 ☐ Yes, frequently
2 ☐ Yes, sometimes
3 ☐ No, rarely
4 ☐ No, never/almost never
5 ☐ I don't have a manager

51. Do you become involved in one or more conflicts at your place of work?

1 ☐ Yes, frequently
2 ☐ Yes, sometimes
3 ☐ No, rarely
4 ☐ No, never/almost never

52. Do you worry that:
Mark with an 'x' in each row.

		Not at all 1	2	3	4	5	6	To a very high degree 7
a.	…your place of work will be reorganised?	☐	☐	☐	☐	☐	☐	☐
b.	…cost-savings or downsizing will affect your job?	☐	☐	☐	☐	☐	☐	☐
c.	…new technology will be introduced in your place of work?	☐	☐	☐	☐	☐	☐	☐
d.	…your will not cope with the work?	☐	☐	☐	☐	☐	☐	☐
e.	…will become unemployed?	☐	☐	☐	☐	☐	☐	☐
f.	…you are a victim of bullying?	☐	☐	☐	☐	☐	☐	☐
g.	…you are a victim of sexual harassment?	☐	☐	☐	☐	☐	☐	☐

53. Do you sometimes feel aversion when you go to work?

1 ☐ Not at all
2 ☐ Rarely
3 ☐ A couple of days a month
4 ☐ One day a week
5 ☐ A couple of days a week
6 ☐ Every day

54. Does bullying take place at your place of work?

1 ☐ Yes
2 ☐ No → **If No, go to section E**

55. Have you been a victim of bullying yourself, at your place of work?

1 ☐ Yes, sometimes
2 ☐ Yes, repeatedly

3 ☐ No

E. Family Affairs and Friends

56. How many people are there in your household?

☐☐ people

57. Are you in a partner relationship?

1 ☐ Yes
2 ☐ No

58. Irrespective of whether you are in a partner relationship or not, to what degree are you satisfied with the way you live?

1 ☐ To a very high degree
2 ☐ To a high degree
3 ☐ Partly
4 ☐ To a slight degree
5 ☐ To a very slight degree

If you do not live in a partner relationship, go to question 60.

59. Compared to other pairs of your acquaintance, to what degree to do you think your partner relationship is on an equal footing?

1 ☐ To a very high degree
2 ☐ To a high degree
3 ☐ Partly
4 ☐ To a slight degree
5 ☐ To a very slight degree

The following questions concern children living at home (biological, adopted, foster children, children of husband/wife/partner).

60. Are there any children living at home?
Count any children who live at least half of the time with you.

1 ☐ Yes
2 ☐ No → *If No,* ***go to question 61***

If Yes: **How many children?**

☐☐ 0 - 5 years old

☐☐ 6 - 12 years old

☐☐ 13 - 17 years old

☐☐ 18 years old or older

61. Are you pregnant?

1 ☐ Yes
2 ☐ No → *If No, **go to question 62***

If Yes: **In which week of pregnancy are you?**

☐☐ weeks

The following questions concern the details of housework and other work at home (also concerns care/supervision/activities related to children, the aged, sick relatives or relations). If you live in a single person household, go to question 65.

62. Who plans most of the housework and other work at home?

1 ☐ I plan the most
2 ☐ My partner plans the most
3 ☐ Another adult in the household plans the most
4 ☐ My partner and I share the planning roughly equally
5 ☐ Another adult in the household and I share the planning roughly equally
6 ☐ Other distribution of the planning

63. How is the housework and other work at home shared out?

1 ☐ I do the most
2 ☐ My partner does the most
3 ☐ Another adult in the household does the most
4 ☐ My partner and I share the work roughly equally
5 ☐ Another adult in the household and I share the work roughly equally
6 ☐ Other distribution of the work
7 ☐ I/we purchase domestic services for most of the work

64. To what degree are you satisfied with the distribution of the housework and other work at home?

1 ☐ To a very high degree
2 ☐ To a high degree
3 ☐ Partly
4 ☐ To a slight degree
5 ☐ To a very slight degree

If you do not have paid work, go to question 68.

65. To what degree to you experience a conflict between your paid work and housework or other work at home?

1 ☐ To a very high degree

2 ☐ To a high degree
3 ☐ Partly
4 ☐ To a slight degree
5 ☐ To a very slight degree

66. To what degree is your housework and other work at home negatively affected by the demands of your work?

1 ☐ To a very high degree
2 ☐ To a high degree
3 ☐ Partly
4 ☐ To a slight degree
5 ☐ To a very slight degree

67. To what degree is your work negatively affected by the demands of your housework and other work at home?

1 ☐ To a very high degree
2 ☐ To a high degree
3 ☐ Partly
4 ☐ To a slight degree
5 ☐ To a very slight degree

68. Sometimes one needs help and support from someone. Do you have a relative or friend who is willing to help if

Mark with a cross on each row.

		Yes<br>1	No<br>2	Do not know<br>3
a.	…you are sick?	☐	☐	☐
b.	…you want company?	☐	☐	☐
c.	…you want to talk with someone about personal problems?	☐	☐	☐
d.	…if you need to borrow 15,000 SEK?	☐	☐	☐

69. Do you sometimes feel alone?

1 ☐ Yes, frequently
2 ☐ Yes, sometimes
3 ☐ No, rarely
4 ☐ No, never/almost never

F. Life Events

70. **Have you during the past twelve months been involved in any of the following? Check** *as many as apply*

1 ☐ Married or entered into consensual union
1 ☐ Had children or adopted children
1 ☐ Divorce or separation
1 ☐ Changed the town where you live
1 ☐ Had children with serious problems (e.g. addiction, criminality, victim of bullying)
1 ☐ Had children who have been the victim of sexual assault
1 ☐ Changed place of work
1 ☐ Received less responsibility at work
1 ☐ Received increased responsibility at work
1 ☐ Experienced seriously worsened finances
1 ☐ Became unemployed
1 ☐ Became homeless
1 ☐ Had serious conflicts with someone close to you
1 ☐ Had serious conflict with someone else
1 ☐ Suffered a serious illness/accident
1 ☐ Has serious illness/accident in the family of among friends
1 ☐ Had someone die in the family or among friends
1 ☐ Other important event(s)
State which, write in the box:
1 ☐ None of the above

The questions below concern the violence that can arise in a close relationship i.e. between spouses/partners or a previous partner, whether one is living together or separately. Where it says partner, we mean husband/wife/partner/partner you live with/partner who lives elsewhere/boyfriend or girl friend. If you do not have a partner, then consider the last relationship you had.

71. **Has a partner**

a) Threatened to hurt you or someone close to you?

1 ☐ No
2 ☐ Yes, 1-2 times
3 ☐ Yes, 3-5 times
4 ☐ Yes, more than 5 times

b) Subjected you to physical and/or sexual violence?

1 ☐ No
2 ☐ Yes, 1-2 times
3 ☐ Yes, 3-5 times
4 ☐ Yes, more than 5 times

72. **Have you, yourself, on any occasion subjected your partner/previous partner to any of these during the past 12 months?**

1 ☐ No
2 ☐ Yes, threat of violence
3 ☐ Yes, physical/sexual violence

73. **Is/was your partner/previous partner a**

1 ☐ Man
2 ☐ Woman

G. Culture and Leisure

74. **How often have you done any of the following during the past 12 months?**
Mark with an 'x' in each row.

		At least once a week<br>1	At least once a month<br>2	At least once a quarter<br>3	Very rarely or never<br>4
a.	Went to the cinema/theatre/museum	☐	☐	☐	☐
b.	Photographed/filmed	☐	☐	☐	☐
c.	Drew/painted	☐	☐	☐	☐
d.	Wrote e.g. diary/poem/articles/blog	☐	☐	☐	☐
e.	Read a book/listened to audio book	☐	☐	☐	☐
f.	Computer/Video game	☐	☐	☐	☐
g.	Went to a concert	☐	☐	☐	☐
h.	Listened to music	☐	☐	☐	☐
i.	Played a musical instrument	☐	☐	☐	☐
j.	Sang in a choir	☐	☐	☐	☐
k.	Went to a sports event as a spectator	☐	☐	☐	☐
l.	Participated in a sport/athletics/other training	☐	☐	☐	☐
m.	Went out in the country	☐	☐	☐	☐
n.	Participated in study circles/courses	☐	☐	☐	☐
o.	Participated in a religious service	☐	☐	☐	☐
p.	Participated in activities of an association	☐	☐	☐	☐
q.	Conversed with friends	☐	☐	☐	☐
r.	Practised some form of textile/woodwork/metalwork or other handicraft	☐	☐	☐	☐
s.	Practised another form of hobby	☐	☐	☐	☐

State where, write in the box:

75. How often do you take some type of exercise (such as run, cycle or train in another way - so the "sweat runs") that lasts for at least 30 minutes a time?

1 ☐ 4 or more time a week
2 ☐ 2-3 times a week
3 ☐ Once a week
4 ☐ A couple of times a month
5 ☐ Never

76. To what degree are you satisfied with your cultural and leisure activities?

1 ☐ To a very high degree
2 ☐ To a high degree
3 ☐ Partly
4 ☐ To a slight degree
5 ☐ To a very slight degree

77. Do you sometimes have difficulty killing time during your spare time?

1 ☐ Yes, frequently
2 ☐ Yes, sometimes
3 ☐ No, rarely
4 ☐ No, never/almost never

H. Alcohol and Smoking Habits

Alcohol and smoking are factors that can be important for an individual's well-being. Therefore, the following questions concern alcohol and smoking habits. To make it easier for you to answer, we show below what is meant by a "standard glass".

A "standard glass" means

spirits fortified wine table wine strong beer medium beer cider

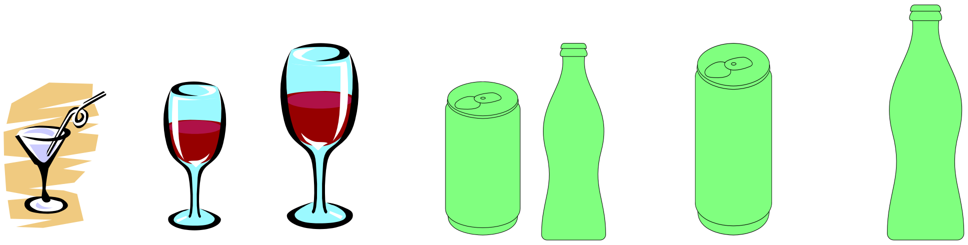

4 cl 8 cl 14 cl 27 cl 45 cl 34 cl

78. Have you at any time during the past 12 months drunk at least one glass of alcohol?

1 ☐ Yes
2 ☐ No → **If No, go to question 86**

79. When you look back over the past 12 months, how regularly have you drunk alcoholic drinks?

1 ☐ Less than once a month
2 ☐ 1-3 times a month
3 ☐ 1-2 times a week
4 ☐ Several times a week
5 ☐ Daily or almost daily

80. How many "standard glasses" do you drink on a typical day when you drink alcohol? (See example above)

1 ☐ 1-2
2 ☐ 3-4
3 ☐ 5-6
4 ☐ 7-9
5 ☐ 10 or more

81. Mark the boxes that are applicable to you with an 'x'.
Mark an 'x' in each row.

		Never<br>1	More rarely than once a month<br>2	Every month<br>3	Every week<br>4	Daily or almost daily<br>5
a.	How often do you drink six "standard glasses" or more on the same occasion)	☐	☐	☐	☐	☐
b.	How often during the past 12 months, have you been unable to stop drinking after you started?	☐	☐	☐	☐	☐
c.	How often during the past 12 months, have you left something undone that you should have done because you drank?	☐	☐	☐	☐	☐
d.	How often during the past 12 months, have you needed a "drink" in the morning after drinking too much the day before?	☐	☐	☐	☐	☐
e.	How often during the past 12 months, have you felt guilty or remorseful due to your drinking?	☐	☐	☐	☐	☐
f.	How often during the past 12 months, have you drunk so much that you cannot remember what you have said and done the next day?	☐	☐	☐	☐	☐

82. Have you or anyone else been injured because of your drinking?

1 ☐ No
2 ☐ Yes, but not during the past year
3 ☐ Yes, during the past year

83. Has a relative or a friend, a doctor or anyone in the health services been worried about your drinking and suggested that you should reduce it?

1 ☐ No
2 ☐ Yes, but not during the past year
3 ☐ Yes, during the past year

84. How many times have you been on sick leave because you have been drinking, during the past 12 months?

1 ☐ It has never happened
2 ☐ 1-3 times
3 ☐ 4-10 times
4 ☐ More than 10 times

85. How many times have you found it difficult to cope with your work because you have been drinking, during the past 12 months?

1 ☐ I do not have a job
2 ☐ It has never happened
3 ☐ 1-3 times
4 ☐ 4-10 times
5 ☐ More than 10 times

86. a) Do you smoke every day?

1 ☐ Yes
2 ☐ No

If No:
b) Do you sometimes smoke every now and again?

1 ☐ Yes
2 ☐ No

Thank you for answering the questionnaire!
